# Addictive social media use during Covid-19 outbreak: Validation of the Bergen Social Media Addiction Scale (BSMAS) and investigation of protective factors in nine countries

**DOI:** 10.1007/s12144-022-03182-z

**Published:** 2022-05-21

**Authors:** Julia Brailovskaia, Jürgen Margraf

**Affiliations:** Mental Health Research and Treatment Center, Department of Clinical Psychology and Psychotherapy, Ruhr-Universität Bochum, Massenbergstr. 9-13, 44787 Bochum, Germany

**Keywords:** Addictive social media use, Bergen Social Media Addiction Scale (BSMAS), Validation, Protective factors, Covid-19, Cross-national

## Abstract

Since the Covid-19 outbreak, addictive social media use increased in many countries. To better understand this development, a universal instrument for the assessment of addictive social media use is required. Against this background, we examined the psychometric properties of the Bergen Social Media Addiction Scale (BSMAS) in representative population samples in nine countries (*N* = 9418, age range: “18 to 24 years” (youngest group), “55 years and older” (oldest group): China, France, Germany, Poland, Russia, Spain, Sweden, U.K., U.S.). Furthermore, we investigated potential factors and mechanisms that could be associated with addictive social media use. Our cross-national findings show that the BSMAS is a unidimensional reliable and valid instrument. Moreover, they reveal that the negative association between positive mental health and addictive social media use is mediated by sense of control in seven of the nine countries (exception: China, Russia). Thus, it can be hypothesized that activities which increase positive mental health could indirectly contribute to the decrease of addictive social media use. We identified conscious engagement in physical activity and a regular sleep rhythm during the pandemic as such potential activities. The fostering of both by governmental programs could enhance positive mental health and reduce addictive social media use.

## Introduction

The outbreak of the coronavirus disease in December 2019 (Covid-19) and its rapid spread during 2020 and 2021 have significantly changed people’s everyday life (World Health Organization, 2021). To slow down the pandemic spread, governments and authorities all over the globe introduced restrictive behavior measures that reduce physical contact among the population (so-called “social distancing”) (Howard et al., 2021; Sohrabi et al., 2020). The measures ranged between the wearing of face masks and maintaining of physical distance to other people in public places, “stay-at-home” requests, and overnight or total curfews (Gandhi & Rutherford, 2020; Tso & Cowling, 2020). The need for “social distancing” resulted in the transfer of many activities and especially social interactions into the online world (Elhai et al., 2021; Lemenager et al., 2021). As a consequence, the popularity of social media such as Facebook, Instagram, TikTok, Snapchat, and Twitter that main aim is to connect people around the globe further increased (DataReportal, 2021; Drouin et al., 2020; Nekliudov et al., 2020; Zhong et al., 2021).

Following available literature, intensive social media use and positive emotions experienced online can foster the development of problematic or addictive tendencies (Andreassen et al., 2016). In line with these findings, addictive social media use also increased since the pandemic outbreak (Masaeli & Farhadi, 2021; Zhao & Zhou, 2021). Addictive social media use is characterized by a close emotional bond to the social media that is accompanied by permanent concerns about the online activities and a strong uncontrollable need to stay permanently online despite potential impairment of other areas of life (Andreassen & Pallesen, 2014; Brailovskaia et al., 2020a). Intensive uneasiness accompanies a temporal leave of the online world (Bányai et al., 2017). Six core addiction elements (Griffiths, 2005) define addictive social media use: salience (permanent thinking about social media use), tolerance (enhanced amount of time is spent on social media to experience positive emotions), mood modification (mood improvement by social media use), relapse (reverting to higher amounts of social media use after unsuccessful attempts to decrease the online behavior), withdrawal symptoms (nervousness and feeling unconfortable without social media use), and conflicts (interpersonal problems caused by intensive social media use) (Andreassen et al., 2017; Griffiths, 2005).

Notably, addictive social media use has not been recognized as a formal psychiatric disorder in the International Classification of Diseases (ICD-11; World Health Organization, 2018) and in the Diagnostic and Statistical Manual of Mental Disorders (DSM-5; American Psychiatric Association, 2013). And similar to other forms of addictive behavior, there is a critical debate about the significance of addictive social media use (Carbonell & Panova, 2017). Some researchers emphasize that it is important not to over pathologize intensive social media use and not to overestimate its potential consequences (Billieux et al., 2015; Carbonell & Panova, 2017; Orben & Przybylski, 2019). In contrast, others warn of the addictive tendencies and their potential negative impact on mental health (Andreassen et al., 2017; Brand et al., 2019; Kircaburun et al., 2020a; Marino et al., 2018). They justify their conclusion by recent international findings from cross-sectional, longitudinal, and experimental studies.

For example, previous cross-sectional research from countries such as China, Germany, Iran, Italy, Lithuania, Poland, and the U.S. described a positive link between addictive social media use and negative factors of mental health such as symptoms of stress, depression, and anxiety (Atroszko et al., 2018; Boursier et al., 2020; Brailovskaia et al., 2021c; Dempsey et al., 2019; Keles et al., 2020; Lin et al., 2017; Worsley et al., 2018). Moreover, longitudinal results from Germany revealed that addictive social media use is positively associated with depression symptoms up to six weeks later (Brailovskaia et al., 2019). In two further independent samples from Germany, addictive social media use was positively linked to suicide-related outcomes (suicidal ideation and suicidal behavior) up to one year later (Brailovskaia et al., 2021a; Brailovskaia et al., 2020c). The relationship between addictive social media use and positive factors such as positive mental health – that is social, emotional and psychological well-being (Lukat et al., 2016) – and life satisfaction was negative (Błachnio et al., 2016; Brailovskaia et al., 2020c, d; Longstreet & Brooks, 2017; Worsley et al., 2018). In three experimental studies from Denmark, Germany, and the U.S., conscious reduction or a complete waiving of social media use significantly reduced addictive tendencies and/or improved mental health (Brailovskaia et al., 2020b; Hunt et al., 2018; Tromholt, 2016).

The presented results emphasize the potential impact of addictive social media use on people’s life and the importance to investigate mechanisms underlying its development and maintenance. Gained knowledge can be used to clarify the current critical debate on the significance of addictive social media use (Carbonell & Panova, 2017). Furthermore, it can contribute to the identification of individuals at risk for the problematic tendencies and the development of prevention programs to protect mental health. This issue gained specific importance since the outbreak of Covid-19. The current increase of addictive social media use (Masaeli & Farhadi, 2021; Zhao & Zhou, 2021) is accompanied by an enhancement of symptoms of stress, depression, and anxiety (Charles et al., 2021; Galea et al., 2020; Liu et al., 2020; Nekliudov et al., 2020; Paredes et al., 2020; Taylor et al., 2020). Considering the described interplay between social media use and mental health (e.g., Gao et al., 2020; Pontes et al., 2018; Xie & Karan, 2019), the understanding of involved mechanisms could provide ways how the negative mental health consequences of the pandemic could be reduced.

Against this background, it is important to overcome current critique on available investigations of addictive social media use. Among others, the critical considerations refer to the lack of a standardized instrument for its assessment that impedes the comparability of available findings (Billieux et al., 2015; Carbonell & Panova, 2017). In the early investigation stage of addictive online behavior, many authors developed own scales for its measurement. In the past years, however, the Facebook Intrusion Questionnaire (FIQ; Elphinston & Noller, 2011) and the short version of the Bergen Facebook Addiction Scale (BFAS; Andreassen et al., 2012) became the preferred instruments for the assessment of addictive social media use (see Duradoni et al., 2020; Marino et al., 2018; Ryan et al., 2014). But their specific focus on Facebook only that limits the generalization to other social media has been repeatedly critiqued (see Bányai et al., 2017; Lin et al., 2017; Monacis et al., 2017). Therefore, the need for an instrument that focuses on social media in general and that includes a more accurate wording and terminology has been emphasized (Griffiths, 2013).

In this vein, Andreassen et al. (2016) who worked with a large Norwegian sample presented the Bergen Social Media Addiction Scale (BSMAS) that assesses addictive tendencies of social media use more generally and thus overcomes the mentioned limitation. The psychometric properties of the BSMAS have been investigated in several countries such as Hungary (Bányai et al., 2017), Iran (Lin et al., 2017), Italy (Monacis et al., 2017), Hong Kong, and Taiwan (Leung et al., 2020) in the past years. All the investigations reported a unidimensional factor structure and satisfying to good scale validity and reliability. Most of the available studies worked with young student samples from one (e.g., Lin et al., 2017; Monacis et al., 2017) or two countries (Leung et al., 2020). Notably, a young sample composition limits the representativity of the results for the general population. This and the lack of cross-national findings that might provide universal evidence are often critiqued in the controversial debate on the significance of addictive social media use (Billieux et al., 2015; Carbonell & Panova, 2017; Orben, 2020).

Therefore, to contribute to the solving of this debate, the first aim of our present study was to investigate the psychometric properties of the BSMAS in population representative samples from nine countries at once. The present study is part of the ongoing international “Bochum Optimism and Mental Health”-Project that investigates risk and protective factors of mental health. Using the framework of our previous comparisons of countries with different welfare systems (Margraf et al., 2021; Margraf et al., 2020b; Scholten et al., 2018; Scholten et al., 2017b), we examined samples from the following nine countries: China, France, Germany, Poland, Russia, Spain, Sweden, the U.K., and the U.S.

Considering the enhancement of addictive social media use since the Covid-19 outbreak (Masaeli & Farhadi, 2021) and earlier findings that described potential negative consequences of problematic online activity (Błachnio & Przepiorka, 2019; Brailovskaia et al., 2019), the second aim of our study was to investigate potential factors and mechanisms that could be linked to addictive social media use. While most available research focused on risk factors that might foster the development of addictive use tendencies (see Marino et al., 2018), we investigated potential protective factors that could reduce addictive social media use.

Specifically, positive mental health is a well-known protective factor that buffers the impact of negative experiences and reduces the risk of mental disorders (e.g., Brailovskaia et al., 2020c, d; Cai et al., 2017; Hu et al., 2020; Teismann et al., 2018; Truskauskaite-Kuneviciene et al., 2020). Among others, positive mental health can foster one’s sense of control (Seligman, 1972) and thus can confer resilience (Brailovskaia & Margraf, 2020; Teismann et al., 2018). Sense of control is an essential element of human beings (Seligman, 1972). Its lack – that is a typical characteristic of people with low levels of positive mental health – can contribute to enhanced levels of helplessness and the strong wish to gain the control back (Skaff, 2007; Southwick & Southwick, 2018). As a consequence, people with low sense of control often tend to dysfunctional coping strategies such as excessive alcohol use and excessive use of media (Kircaburun et al., 2020b; Volpicelli, 1987). Some of them try to compensate the loss of control in the offline world by online activity (Brailovskaia et al., 2021b). In the short-term, this strategy may contribute to positive emotions and to the forgetting of negative ones. To enhance the positive experiences, the individual often continues the excessive online behavior (Sun & Zhang, 2020; Zhao & Zhou, 2021). However, following the Interaction of Person-Affect-Cognition-Execution (I-PACE) model for addictive behavior, in the longer-term, due to the interaction between different moderating (e.g., general inhibitory control) and mediating (e.g., affective and cognitive responses to triggers) factors, the excessive online activity can contribute to the development of addictive tendencies and increase conflicts in the offline world (Brand et al., 2019). Therefore, it is important to identify further factors that can contribute to the addictive tendencies and to understand their interaction.

Considering available literature, positive mental health and sense of control could belong to such factors. Both negatively predicted addictive social media use (Brailovskaia & Margraf, 2021; Brailovskaia et al., 2019). Notably, positive mental health contributes to one’s sense of control (e.g., Seligman, 1972). Thus, it might be that sense of control mediates the relationship between positive mental health and addictive social media use. Specifically, high levels of positive mental health could be associated with high sense of control, and high sense of control could be related to low addictive social media use. Recent research contributes to this assumption. Positive mental health assessed before the pandemic outbreak, predicted sense of control after the outbreak (Brailovskaia & Margraf, 2020). Furthermore, people with low sense of control over important areas of their everyday life due to the Covid-19 situation were shown to be at risk for enhanced levels of addictive online activity (Brailovskaia & Margraf, 2021).

**Fig. 1:**
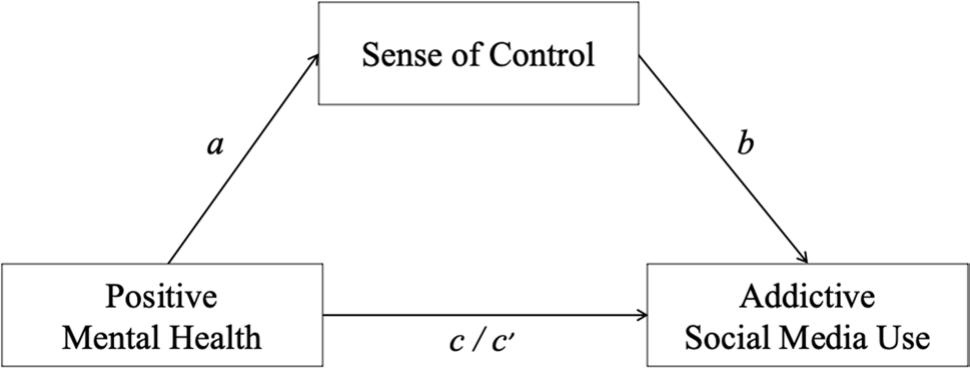
Hypothesized median model with positive mental health (predictor), sense of control (mediator), and addictive social media use (outcome). *Note.* Path *a*: link between positive mental health and sense of control; path *b*: link between sense of control and addictive social media use; path *c* (the total effect): basic relationship between positive mental health and addictive social media use; path *c’* (the direct effect): link between positive mental health and addictive social media use after the inclusion of sense of control in the model

Against the presented background, we hypothesized that addictive social media use – as assessed with the BSMAS – is negatively associated with positive mental health (Hypothesis 1a) and with sense of control (Hypothesis 1b). Positive mental health and sense of control were expected to be positively associated (Hypothesis 1c). Moreover, we hypothetically assumed that sense of control could mediate the relationship between positive mental health and addictive social media use (Hypothesis 1d). Figure 1 visualizes the hypothesized mediation model. The investigation of Hypothesis 1 at the same time in all nine countries could reveal whether positive mental health and sense of control might be universal protective factors against addictive social media use.

In addition to the hypothesized protective impact of positive mental health on addictive social media use, recent research described that positive mental health is negatively associated with psychological burden caused by the Covid-19 situation. Moreover, it could contribute to the adherence to behavioral governmental measures that are necessary to reduce the pandemic spread (Brailovskaia & Margraf, 2020; Margraf et al., 2020a). Therefore, our third aim was to investigate factors that might contribute to the enhancement of positive mental health especially during the pandemic. The findings could also indirectly reveal how to reduce addictive social media use (i.e., by the increase of positive mental health; see Hypothesis 1).

Notably, regular engagement in moderate physical activity (e.g., jogging, cycling, yoga) is recommended by the World Health Organization (2010) to enhance mental and physical health. It can reduce the negative impact of stressful life events, foster the fading out of negative emotions and the experience of positive emotions (Eime et al., 2013; Wang et al., 2012). Therefore, we hypothesized that conscious engagement in regular physical activity during the pandemic might be positively linked to positive mental health (Hypothesis 2a). Moreover, a regular sleep rhythm is a well-known protective factor of mental health (Velten et al., 2014), while an irregular sleep rhythm can foster depression and anxiety symptoms (Anderson & Bradley, 2013; Buysse et al., 2008). In a longitudinal study, the regularity of the sleep rhythm was positively linked to positive mental health (Cai et al., 2017). Considering this background, we assumed that a consciously regular sleep rhythm during the pandemic might be positively associated with one’s positive mental health (Hypothesis 2b). Thus, the investigation of Hypothesis 2 should reveal whether conscious engagement in physical activity and a consciously regular sleep rhythm could foster positive mental health in the current extraordinary Covid-19 situation.

The hypotheses were examined separately in each sample in order to investigate whether the same result pattern can be found in the nine included countries or whether we can identify possible country-specific differences.

## Methods

### Procedure and Participants

The overall investigated sample was comprised of 9418 participants from nine countries: China: *N* = 1030; France: *N* = 1012, Germany: *N* = 1175, Poland: *N* = 1012, Russia: *N* = 1020, Spain: *N* = 997, Sweden: *N* = 1022, the U.K.: *N* = 1073, and the U.S.: *N* = 1077. Table 1 shows the demographics of the country-specific samples. Data were collected within eight days (May 12 to May 19, 2021) by an independent social marketing and research institute (YouGov, www.yougov.de) via population-based online-panel surveys in the national language of the countries. Participants were recruited from residential populations aged 18 years and above. Age, gender, and region stratification were implemented to achieve representativeness. In all countries, participation was compensated by panel-specific tokens that can be converted in voucher or monetary payment. We focused on addictive social media use and the validation of the BSMAS. Therefore, the only requirement for participation was the general use of any form of social media. All participants met this requirement. The responsible Ethics Committee approved the current study’s implementation. YouGov obtained all required permits and approvals for the datal collection in the nine countries. There were no missing data. All datasets were complete. The study is part of a larger cross-national investigation of the “Bochum Optimism and Mental Health”-Project. It was pre-registered with AsPredicted.org on May 05, 2021 (Pre-registration Number: #64865). All participants were properly instructed and gave online their informed consent to participate. All national regulations and laws regarding human subjects research were followed. The study was conducted in accordance with the Declaration of Helsinki.

**Table 1:** Demographic variables in the country-specific samples

	China	France	Germany	Poland	Russia	Spain	Sweden	U.K.	U.S.
Gender (female, %)	44.5	54.9	52.5	53.7	51.5	51.9	51.7	55.4	51.3
Age groups (%)									
18 to 24 years	25.4	8.9	8.2	9.8	8.6	7.6	5.9	2.9	8.6
25 to 34 years	35.7	15.0	14.5	18.0	21.6	14.2	20.0	13.9	13.6
35 to 44 years	23.3	16.4	14.4	19.5	21.7	20.1	12.9	14.9	14.9
45 to 54 years	11.3	17.2	19.7	15.6	18.4	20.4	17.1	15.6	18.2
55 years and older	4.3	42.5	43.3	37.2	29.7	37.7	44.1	53.0	44.7
Marital Status (%)									
Single	37.3	23.5	26.0	22.0	14.0	23.1	34.2	21.2	23.2
Romantic relationship, not married	10.8	22.9	15.0	16.8	13.6	20.0	21.2	15.0	7.9
Married	48.7	40.1	43.6	48.9	57.5	44.7	35.3	49.5	54.6
Widowed, divorced	3.2	13.4	15.4	12.3	14.9	12.2	9.2	14.4	14.3
Living Environment (%)									
Large city	42.9	29.7	35.1	44.1	74.9	38.9	45.5	22.4	35.7
Small city	44.4	39.3	37.4	33.6	19.7	41.1	33.3	35.6	42.9
Rural community	12.7	30.9	27.5	22.3	5.4	20.0	21.2	42.0	21.4

China: *N* = 1030, France: *N* = 1012, Germany: *N* = 1175, Poland: *N* = 1012, Russia: *N* = 1020, Spain: *N* = 997, Sweden: *N* = 1022, U.K.: *N* = 1073, U.S.: *N* = 1077; due to rounding, the sum of the frequencies is not always 100%

### Measures

#### Demographics

Participants were asked to indicate their gender, age range, marital status, and living environment (see Table 1).

#### Time Spent on Social Media Use

Participants were asked to provide the daily time they spent on social media use in minutes (i.e., “On average, how much time per day do you spend using social media (e.g., Facebook, Instagram, Twitter, etc.)?”).

#### Addictive Social Media Use

The Bergen Social Media Addiction Scale (BSMAS; Andreassen et al., 2016) assessed addictive social media use. The content of the six items refers to the course of the last year (i.e., “How often during the last year have you…”). Each item reflects one of the six core addiction elements (Griffiths, 2005). For example: “…spent a lot of time thinking about social media or planned use of social media?” (i.e., salience), “...felt an urge to use social media more and more?” (i.e., tolerance), and “...become restless or troubled if you have been prohibited from using social media?” (i.e., withdrawal) (Andreassen et al., 2016; Andreassen et al., 2017). The items are rated on a 5-point Likert-type scale (1 = *very rarely*, 2 = *rarely*, 3 = *sometimes*, 4 = *often*, 5 = *very often*). The higher the sum score, the higher the level of addictive social media use. The overall sum score can range from 6 to 30. Current scale reliability of the BSMAS ranged between Cronbach’s *α* = .815 (China) and *α* = .901 (Germany).

#### Positive Mental Health

The unidimensional Positive Mental Health Scale (PMH-Scale; Lukat et al., 2016) assessed the level of positive mental health. The PMH-Scale is an internationally well-established instrument for the assessment of psychological, emotional, and social well-being. The PMH-Scale includes nine items that are rated on a 4-point Likert-type scale (e.g., “I enjoy my life”; 0 = *do not agree*, 3 = *agree*). The higher the sum score, the higher the level of positive mental health. The total sum score can range from zero to 27. Current scale reliability is *α* = .895 (France) to .937 (Sweden).

#### Depression, Anxiety, and Stress Symptoms

The Depression Anxiety Stress Scales 21 (DASS-21; Lovibond & Lovibond, 1995) measured symptoms of depression, anxiety and stress with respectively seven items per subscale (e.g., depression subscale: “I felt that life was meaningless”; anxiety subscale: “I felt scared without any good reason”; stress subscale: “I found it hard to wind down”). Items are rated on a 4-point Likert-type scale (0 = *did not apply to me at all*, 3 = *applies to me very much or most of the time*). Higher sum scores indicate higher symptoms of depression, anxiety, and stress. The total sum score of each subscale can range from zero to 21. Current scale reliability of depression subscale: *α* = .899 (China) to .943 (the U.K.); of anxiety subscale: *α* = .864 (Germany) to .898 (Russia, the U.S.); and of stress subscale: *α* = .877 (China) to .919 (France, Germany, Poland).

#### Sense of Control

Following Niemeyer et al. (2019) sense of control was assessed by the two-item scale (i.e., Item 1: “Do you experience important areas of your life (i.e., work, free-time, family, etc.) to be uncontrollable, meaning that you cannot, or barely can, influence them?”; Item 2: “Do you experience these important areas of your life as unpredictable or inscrutable?”). Both items are rated on a 5-point Likert-type scale (0 = *not at all*, 4 = *very strong*). We reversed the items for the sum score calculation. Higher sum scores indicate higher sense of control. The total sum score can range from zero to eight. Current scale reliability ranges between *α* = .790 (Poland) and .912 (Spain).

#### Conscious Physical Activity and Sleep Rhythm

Participants were asked to rate their agreement with the statements “Compared to the time before the Covid-19 outbreak, I consciously do more physical activity” and “Compared to the time before the Covid-19 outbreak, my sleep rhythm has consciously become more regular” (0 = *I do not agree*, 1 = *I do agree*).

When available, previously validated national language versions of the used scales were included (e.g., BSMAS: Andreassen et al., 2017; Brailovskaia et al., 2020a; PMH-Scale: Cai et al., 2017; Margraf, Brailovskaia, et al., 2020; Sense of Control Scale: Niemeyer et al., 2019; DASS-21: Scholten et al., 2017a). In case that no previously validated national language version was available, the measures were translated into the national language from the English language version by the customary translation-back-translation-modification procedure (Berry, 1989) by the international team of the “Bochum Optimism and Mental Health”-Project.

### Statistical Analyses

Statistical analyses were conducted using the Statistical Package for the Social Sciences (SPSS 26; IBM Corp., 2019) and the macro Process version 3.5 (www.processmacro.org/index.html; Hayes, 2017). The distribution scores of all assessed scales were close to a normal distribution (indicated by analyses of skewness and kurtosis). In the first step, we investigated the psychometric properties of the BSMAS. Available research from countries such as Hungary (Bányai et al., 2017), Iran (Lin et al., 2017), Italy (Monacis et al., 2017), Hong Kong, and Taiwan (Leung et al., 2020) reported a unidimensional structure for the BSMAS. However, the structure of the BSMAS has not been previously investigated in most of the nine countries included in the present investigation. Therefore, we decided not to postulate a specific factor structure by calculating a confirmatory factor analysis (CFA). Instead, we ran an exploratory factor analysis (EFA) using principal component analysis (PCA; rotation method: varimax) on the six items of the BSMAS to investigate the underlying structure without any biases (Field, 2013; Schmitt, 2011). Then, we assessed the scale properties of the BSMAS by the calculation of internal consistency (Cronbach’s *α*), mean interitem correlation (*r*_mi_), item-total scale correlation (*r*_it_), and item difficulty (*p*_m_). To investigate the construct validity of the BSMAS, the associations of addictive social media use with time daily spent on social media, depression, anxiety, and stress symptoms (convergent validity), as well as with positive mental health and sense of control (discriminant validity) were assessed by the calculation of zero-order bivariate correlation analyses (Bühner, 2011).

In the second step, the hypothesized mediation effect was investigated. Therefore, first, the relationship between positive mental health and sense of control was assessed by a zero-order bivariate correlation analysis. Next, a mediation analysis (model 4) tested the association between positive mental health (predictor), sense of control (mediator), and addictive social media use (outcome), controlling for the demographic variables (gender (0 = *woman*, 1 = *man*), age group, marital status, living environment) and daily social media use time as covariates. The basic relationship between positive mental health and addictive social media use was denoted by path *c* (the total effect). Path *a* denoted the link between positive mental health and sense of control, and path *b* denoted the relationship between sense of control and addictive social media use. The combined effect of path *a* and path *b* represented the indirect effect. The link between positive mental health and addictive social media use after the inclusion of sense of control in the model was denoted by path *c’* (the direct effect) (see Fig. 1). The bootstrapping procedure (10.000 samples) providing percentile bootstrap confidence intervals (*CI* 95%) assessed the mediation effect.

In the third step, a two-step hierarchical regression analysis was calculated that included positive mental health as outcome. Step 1 included the demographics (as control variables); Step 2 included physical activity and sleep rhythm (as statistical predictors). There was no violation of the multi-collinearity assumption (all values of tolerance > .25, all variance inflation factor values <5; Urban & Mayerl, 2006).

All calculations were conducted in the nine country-specific samples, respectively.

## Results

### Factor Structure of the BSMAS

The EFA replicated the unidimensional factor structure of the BSMAS in all nine country-specific samples (Kaiser-Meyer-Olkin: KMO = .810 (Spain) to .877 (Germany, Sweden); Barlett’s test of sphericity: *χ*^*2*^ = 2689.658 (Spain) to *χ*^*2*^ = 4236.070 (Germany), all: df = 15, *p* < .001). The eigenvalue of the factor ranged between 3.130 (China) and 4.048 (Germany), and it explained 52.2% (China) to 67.5% (Germany) of the variance. As shown in Table 2 and in Table 3, the factor loadings were sufficient for all six items. They ranged for Item 1 between .667 (China) and .793 (Sweden), for Item 2 between .747 (China) and .857 (Poland), for Item 3 between .792 (China) and .848 (Germany), for Item 4 between .641 (France) and .808 (Germany), for Item 5 between .694 (Spain) and .847 (Sweden), and for Item 6 between .682 (China) and .821 (Germany). Item 2 was the item with the lowest loading in five of the nine countries (Poland, Russia, Spain, the U.K., the U.S.), while Item 4 was the item with the highest loading in most of the countries (seven of nine: France, Poland, Russia, Spain, Sweden, the U.K., the U.S.).

**Table 2:** Descriptive statistics and properties of the Bergen Social Media Addiction Scale items (part 1)

Country	BSMAS-Item	*M (SD)*	*Min–Max*	*p*_m_	*r*_it_	*α without item*	*EFA load*
China, *N* = 1030	1	2.89 (1.03)	1–5	.577	.514	.799	.667
	2	2.97 (1.01)	1–5	.595	.606	.780	.747
	3	2.66 (1.04)	1–5	.533	.660	.768	.792
	4	2.70 (1.05)	1–5	.539	.587	.784	.728
	5	2.78 (1.10)	1–5	.556	.567	.789	.711
	6	2.68 (1.07)	1–5	.536	.535	.795	.682
France, *N* = 1012	1	1.92 (1.05)	1–5	.383	.679	.836	.786
	2	2.21 (1.19)	1–5	.441	.695	.833	.801
	3	2.06 (1.19)	1–5	.411	.687	.835	.794
	4	2.10 (1.18)	1–5	.420	.513	.867	.641
	5	1.66 (.99)	1–5	.331	.713	.831	.823
	6	1.57 (.91)	1–5	.314	.694	.836	.807
Germany, *N* = 1175	1	2.06 (1.11)	1–5	.411	.475	.891	.778
	2	1.95 (1.14)	1–5	.390	.389	.876	.848
	3	1.85 (1.14)	1–5	.369	.573	.877	.848
	4	1.75 (1.05)	1–5	.350	.532	.886	.808
	5	1.52 (.91)	1–5	.304	.631	.885	.824
	6	1.50 (.88)	1–5	.300	.593	.886	.821
Poland, *N* = 1012	1	2.47 (1.21)	1–5	.493	.690	.870	.791
	2	2.55 (1.25)	1–5	.510	.778	.855	.857
	3	2.42 (1.30)	1–5	.483	.762	.858	.847
	4	2.24 (1.16)	1–5	.448	.594	.884	.708
	5	1.97 (1.13)	1–5	.394	.708	.867	.804
	6	1.97 (1.16)	1–5	.393	.685	.871	.787
Russia, *N* = 1020	1	2.87 (1.28)	1–5	.574	.653	.855	.766
	2	2.73 (1.30)	1–5	.545	.751	.838	.842
	3	2.65 (1.34)	1–5	.531	.747	.838	.840
	4	2.21 (1.17)	1–5	.443	.582	.866	.702
	5	2.22 (1.24)	1–5	.444	.728	.842	.826
	6	1.82 (1.13)	1–5	.365	.588	.865	.707

BSMAS = Bergen Social Media Addiction Scale; *M* = Mean, *SD*=Standard Deviation, *Min* = Minimum, *Max* = Maximum; *r*_it_ = item-total scale correlation; *p*_m_ = item difficulty; *α* = Cronbach’s *α*; EFA load = loading of the items in the exploratory factor analysis

**Table 3:** Descriptive statistics and properties of the Bergen Social Media Addiction Scale items (part 2)

Country	BSMAS-Item	*M (SD)*	*Min–Max*	*p*_m_	*r*_it_	*α without item*	*EFA load*
Spain, *N* = 997	1	2.76 (1.20)	1–5	.551	.654	.815	.777
	2	2.63 (1.25)	1–5	.527	.752	.795	.849
	3	2.61 (1.30)	1–5	.522	.689	.808	.804
	4	2.32 (1.15)	1–5	.464	.539	.837	.673
	5	1.82 (1.08)	1–5	.364	.559	.833	.694
	6	1.69 (1.00)	1–5	.337	.577	.831	.704
Sweden, *N* = 1022	1	2.14 (1.15)	1–5	.428	.704	.884	.793
	2	2.18 (1.17)	1–5	.435	.758	.876	.835
	3	1.99 (1.19)	1–5	.398	.758	.876	.840
	4	1.91 (1.12)	1–5	.381	.689	.886	.788
	5	1.66 (.98)	1–5	.332	.763	.876	.847
	6	1.57 (.95)	1–5	.314	.699	.886	.798
United Kingdom, *N* = 1073	1	1.89 (1.05)	1–5	.378	.658	.846	.761
	2	1.95 (1.09)	1–5	.389	.774	.824	.850
	3	1.79 (1.10)	1–5	.359	.709	.837	.808
	4	1.86 (1.10)	1–5	.371	.611	.856	.731
	5	1.40 (.77)	1–5	.279	.674	.847	.793
	6	1.35 (.74)	1–5	.270	.620	.855	.748
United States, *N* = 1077	1	2.15 (1.21)	1–5	.430	.647	.855	.758
	2	2.18 (1.24)	1–5	.436	.746	.838	.835
	3	2.13 (1.27)	1–5	.426	.688	.848	.794
	4	2.07 (1.21)	1–5	.414	.585	.866	.705
	5	1.74 (1.12)	1–5	.349	.702	.846	.809
	6	1.67 (1.08)	1–5	.335	.683	.850	.793

*BSMAS *= Bergen Social Media Addiction Scale; *M* = Mean, *SD*=Standard Deviation, *Min* = Minimum, *Max* = Maximum; *r*_it_ = item-total scale correlation; *p*_m_ = item difficulty; *α* = Cronbach’s *α*; EFA load = loading of the items in the exploratory factor analysis

### Normal Distribution and Scale Properties of the BSMAS

Analyses of skewness and kurtosis revealed acceptable closeness to a normal distribution for the BSMAS in all nine countries. The skewness ranged between −.032 (China) and 1.102 (the U.K.), the kurtosis ranged between .026 (the U.S.) and .754 (the U.K.). The internal consistency of the BSMAS in the nine countries was good (all countries, except Germany) to excellent (Germany) (Field, 2013): China: *α* = .815, France: *α* = .863, Germany: *α* = .901, Poland: *α* = .887, Russia: *α* = .873, Spain: *α* = .846, Sweden: *α* = .899, the U.K.: *α* = .867, and the U.S.: *α* = .872. In all samples, the respectively exclusion of single items did not provide significant improvement of the internal consistency (see Tables 2 and 3).

The mean interitem correlation was acceptable in China (*r*_mi_ = .424, range: .265 to .584) and in Spain (*r*_mi_ = .476, range: .345 to .768). In all the other countries, it was slightly above the recommended range (i.e., .150 to .500; Briggs & Cheek, 1986; Clark & Watson, 1995): France: *r*_mi_ = .522 (range: .360 to .722), Germany: *r*_mi_ = .609 (range: .491 to .741), Poland: *r*_mi_ = .567 (range: .404 to .760), Russia: *r*_mi_ = .532 (range: .329 to .735), Sweden: *r*_mi_ = .601 (range: .477 to .744), the U.K.: *r*_mi_ = .534 (range: .401 to .727), and the U.S.: *r*_mi_ = .535 (range: .440 to .695).

As shown in Tables 2 and 3, the item-total scale correlations were good (Field, 2013) and showed a similar result pattern as in previous studies (e.g., Leung et al., 2020; Lin et al., 2017). Their range was: China: *r*_it_ = .514 (Item 1) and *r*_it_ = .660 (Item 3); France: *r*_it_ = .513 (Item 4) and *r*_it_ = .713 (Item 5); Germany: *r*_it_ = .389 (Item 2) and *r*_it_ = .631 (Item 5), Poland: *r*_it_ = .594 (Item 4) and *r*_it_ = .778 (Item 2); Russia: *r*_it_ = .581 (Item 4) and *r*_it_ = .751 (Item 2); Spain: *r*_it_ = .539 (Item 4) and *r*_it_ = .752 (Item 2); Sweden: *r*_it_ = .689 (Item 4) and *r*_it_ = .763 (Item 5); the U.K.: *r*_it_ = .611 (Item 4) and *r*_it_ = .774 (Item 2); and the U.S.: *r*_it_ = .585 (Item 4) and *r*_it_ = .746 (Item 2).

The item difficulty was acceptable in all countries (Field, 2013; Lienert & Raatz, 1994). It ranged between (see Tables 2 and 3): China: *p*_m_ = 51.4% (Item 1) and *p*_m_ = 66.0% (Item 3); France: *p*_m_ = 31.4% (Item 6) and *p*_m_ = 44.1% (Item 2); Germany: *p*_m_ = 30.0% (Item 6) and *p*_m_ = 41.1% (Item 1); Poland: *p*_m_ = 39.3% (Item 6) and *p*_m_ = 51.0% (Item 2); Russia: *p*_m_ = 36.5% (Item 6) and *p*_m_ = 57.4% (Item 1); Spain: *p*_m_ = 33.7% (Item 6) and *p*_m_ = 55.1% (Item 1); Sweden: *p*_m_ = 31.4% (Item 6) and *p*_m_ = 43.5% (Item 2); the U.K.: *p*_m_ = 27.0% (Item 6) and *p*_m_ = 38.9% (Item 2); and the U.S.: *p*_m_ = 33.5% (Item 6) and *p*_m_ = 43.6% (Item 2).

The descriptive analysis of the single items revealed that Item 6 (interpersonal conflicts) was the item with the lowest mean score in eight of the nine countries (exception: China: Item 3). Item 2 (tolerance) was the item with the highest mean score in five countries (China, France, Poland, Sweden, the U.S.) and Item 1 (salience) was the item with the highest mean score in the other four countries (Germany, Russia, Spain, the U.K.) (see Tables 2 and 3).

### Construct Validity of the BSMAS

The descriptive statistics of the BSMAS and of the other assessed constructs are presented in Table 4. Table 5 displays the correlations of the BSMAS.

**Table 4:** Descriptive statistics of the investigated variables

	China	France	Germany	Poland	Russia	Spain	Sweden	U.K.	U.S.
*M (SD)*									
Daily Time of Social Media Use (in minutes)	210.69 (147.32)	108.42 (128.82)	82.09 (115.26)	150.58 (145.32)	156.39 (146.91)	138.72 (131.61)	116.40 (120.39)	83.15 (97.34)	121.04 (140.31)
Addictive Social Media Use	16.67 (4.53)	11.51 (5.04)	10.62 (5.13)	13.61 (5.77)	14.51 (5.84)	13.82 (5.26)	11.45 (5.37)	10.24 (4.59)	11.95 (5.59)
Positive Mental Health	16.64 (5.92)	16.23 (5.42)	16.66 (5.93)	17.02 (6.67)	16.37 (5.48)	16.84 (5.66)	16.12 (6.86)	16.42 (5.88)	17.86 (6.03)
Sense of Control	4.60 (1.76)	5.61 (2.15)	5.83 (2.05)	4.85 (2.04)	5.25 (1.95)	5.35 (2.29)	5.65 (2.23)	6.09 (2.05)	6.01 (2.16)
Depression Symptoms	5.45 (4.94)	4.71 (5.39)	5.00 (5.33)	6.55 (5.68)	5.75 (4.99)	6.12 (5.66)	4.91 (5.48)	5.11 (5.47)	5.24 (5.66)
Anxiety Symptoms	5.28 (4.45)	3.68 (4.57)	3.21 (4.05)	5.20 (5.01)	4.58 (4.79)	4.68 (5.03)	3.43 (4.44)	2.90 (3.94)	3.92 (4.84)
Stress Symptoms	6.37 (4.62)	5.59 (5.36)	5.52 (5.16)	7.07 (5.43)	6.70 (5.13)	7.15 (5.24)	5.57 (4.86)	5.27 (4.98)	5.43 (5.17)
Frequency *(yes, %)*									
Physical Activity	72.6	40.7	29.3	46.8	52.5	48.4	36.6	36.1	43.2
Sleep Rhythm	57.7	34.3	33.3	41.5	49.1	37.7	33.7	29.8	42.7

China: *N* = 1030, France: *N* = 1012, Germany: *N* = 1175, Poland: *N* = 1012, Russia: *N* = 1020, Spain: *N* = 997, Sweden: *N* = 1022, U.K.: *N* = 1073, U.S.: *N* = 1077; *M* = Mean; *SD *= Standard Deviation

**Table 5:** Correlation of addictive social media use with time spent on social media use and mental health factors (construct validity of Bergen Social Media Addiction Scale)

	China	France	Germany	Poland	Russia	Spain	Sweden	U.K.	U.S.
Addictive Social Media Use “with”									
Daily Time of Social Media Use	.193**	.482**	.465**	.502**	.434**	.479**	.509**	.539**	.476**
Positive Mental Health	−.033	−.132**	−.160**	− .142**	.013	−.173**	−.180**	−.166**	−.212**
Sense of control	−.341**	−.327**	−.329**	−.315**	−.331**	−.313**	−.362**	−.266**	−.444**
Depression Symptoms	.346**	.423**	.402**	.438**	.413**	.419**	.400**	.256**	.516**
Anxiety Symptoms	.382**	.466**	.467**	.462**	.472**	.477**	.515**	.300**	.622**
Stress Symptoms	.418**	.426**	.420**	.437**	.447**	.481**	.485**	.346**	.570**

China: *N* = 1030, France: *N* = 1012, Germany: *N* = 1175, Poland: *N* = 1012, Russia: *N* = 1020, Spain: *N* = 997, Sweden: *N* = 1022, U.K.: *N* = 1073, U.S.: *N* = 1077; **p < .01

#### Convergent Validity

The mean sum score of the BSMAS ranged between 10.24 (*SD* = 4.59) in the U.K. and 16.67 (*SD* = 4.53) in China. Descriptive analyses of the measures that were expected to be positively linked to the scores of the BSMAS revealed that mean daily time spent on social media use ranged between 82.09 (*SD* = 115.26) minutes in Germany and 210.69 (*SD* = 147.32) minutes in China. The mean sum score of the DASS-21 depression subscale ranged between 4.71 (*SD* = 5.39) in France and 6.55 (*SD* = 5.68) in Poland, of the DASS-21 anxiety subscale between 3.21 (*SD* = 4.05) in Germany and 5.28 (*SD* = 4.45) in China, and of the DASS-21 stress subscale between 5.27 (*SD* = 4.98) in the U.K. and 7.15 (*SD* = 5.24) in Spain. In all nine countries, the scores of the BSMAS were significantly positively correlated with the time spent on social media use (*r* = .193 (China) to *r* = .539 (the U.K.), all: *p* < .01) and with the scores of the DASS-21 depression subscale (*r* = .256 (the U.K.) to *r* = .516 (the U.S.), all: *p* < .01), anxiety subscale (*r* = .300 (the U.K.) to *r* = .622 (the U.S.), all: *p* < .01), and stress subscale (*r* = .346 (the U.K.) to *r* = .570 (the U.S.), all: *p* < .01) (see Table 5). The results reveal a good convergent validity of the BSMAS in the nine investigated countries (Field, 2013).

#### Discriminant Validity

Descriptive analyses of the measures that were expected to be negatively related to the scores of the BSMAS showed that the mean sum score of the PMH-Scale ranged between 16.12 (*SD* = 6.86) in Sweden and 17.86 (*SD* = 6.03) in the U.S. The mean sum score of the Sense of Control Scale ranged between 4.60 (*SD* = 1.76) in China and 6.09 (*SD* = 2.05) in the U.K. (see Table 4). Table 5 shows that the scores of the BSMAS were significantly negatively correlated with positive mental health (*r* = −.132 (France) to *r* = −.212 (the U.S.), all: *p* < .01) in all countries, except China and Russia (both: *p* > .05). Furthermore, the scores of the BSMAS were significantly negatively correlated with sense of control in all nine countries (*r* = −.266 (the U.K.) to *r* = −.444 (the U.S.), all: *p* < .01). The results reveal a good discriminant validity of the BSMAS in France, Germany, Poland, Spain, Sweden, the U.K., and the U.S. The discriminant validity in China and Russia is acceptable (Field, 2013).

#### Correlation and Mediation Analyses: Relationship between Positive Mental Health, Sense of Control and Addictive Social Media Use

**Fig. 2:**
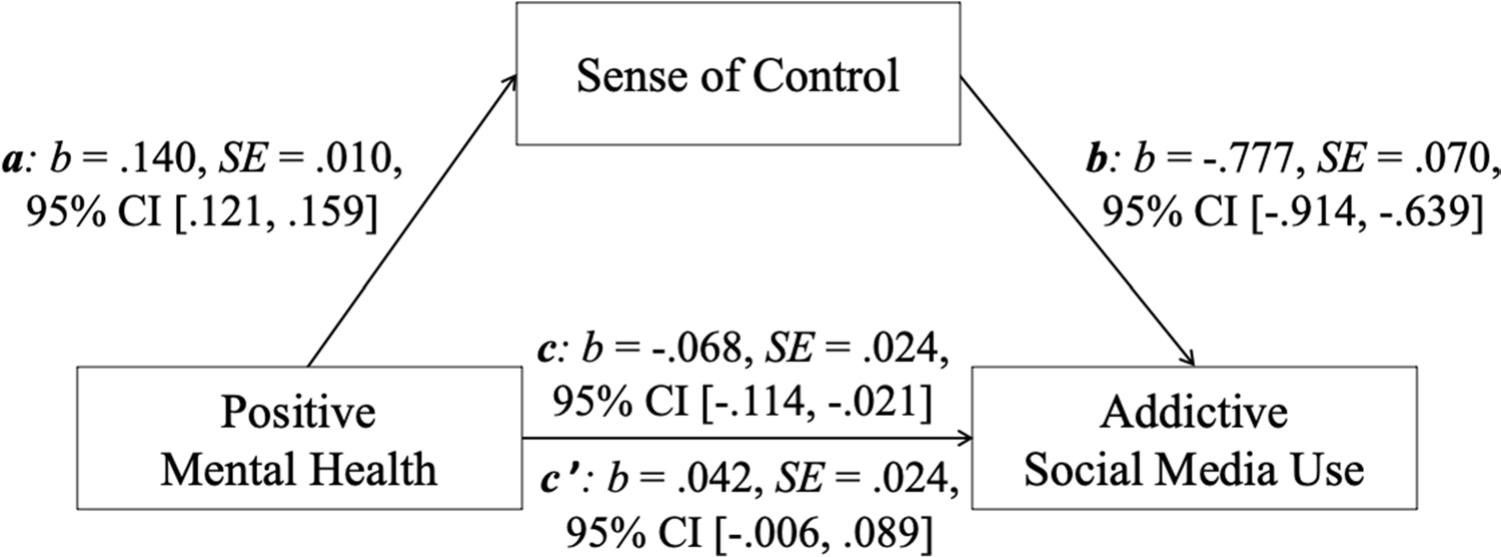
Exemplary presentation of the results of the median model with positive mental health (predictor), sense of control (mediator), and addictive social media use (outcome) in the U.S. sample (*N* = 1077). *Note.* Path *a*: link between positive mental health and sense of control; path *b*: link between sense of control and addictive social media use; path *c* (the total effect): basic relationship between positive mental health and addictive social media use; path *c’* (the direct effect): link between positive mental health and addictive social media use after the inclusion of sense of control in the model

In all nine countries, positive mental health was significantly positively correlated with sense of control: China: *r* = .200, France: *r* = .372, Germany: *r* = .440, Poland: *r* = .365, Russia: *r* = .157, Spain: *r* = .461, Sweden: *r* = .490, the U.K.: *r* = .569, and the U.S.: *r* = .460 (all: *p* < .01). As shown in Table 6, the mediation model was significant in seven of the nine countries (France, Germany, Poland, Spain, Sweden, the U.K., and the U.S.). Sense of control significantly statistically mediated the negative relationship between positive mental health and addictive social media use (full mediation effect). The basic relationship between positive mental health (predictor) and addictive social media use (outcome) was significant (see Table 6, total effect, *c*). The relationship between positive mental health and sense of control (mediator) (path *a*: *p* < .001), as well as the association between sense of control and addictive social media use (path *b*: *p* < .001) were also significant. The link between positive mental health and addictive social media use was not significant after the inclusion of sense of control in the model (see Table 6, direct effect, *c’*). The indirect effect was significant (see Table 6, indirect effect, *ab*). In China and in Russia the mediation model was not significant (see Table 6). Figure 2 visualizes exemplary the mediation results in the U.S. sample.

**Table 6:** Estimated coefficients of the mediation models with positive mental health (predictor), sense of control (mediator), and addictive social media use (outcome)

	Total effect			Direct effect			Indirect effect		
	*c*	*SE*	95% CI	*c’*	*SE*	95% CI	*ab*	*SE*	95% CI
China *(N = 1030)*									
Addictive Social Media Use	−.021	.024	[−.068, .026]	.032	.023	[−.013, .076]	−.053	.011	[−.076, −.033]
France *(N = 1012)*									
Addictive Social Media Use	−.085	.025	[−.134, −.036]	−.021	.026	[−.073, .030]	−.064	.012	[−.089, −.042]
Germany *(N = 1175)*									
Addictive Social Media Use	−.112	.024	[−.159., −.066]	−.026	.026	[−.076, .024]	−.086	.014	[−.116, −.059]
Poland *(N = 1012)*									
Addictive Social Media Use	−.094	.024	[−.141, −.047]	−.006	.024	[−.054, .042]	−.088	.012	[−.112, −.066]
Russia *(N = 1020)*									
Addictive Social Media Use	−.016	.030	[−.075, .043]	.032	.029	[−.025, .090]	−.048	.011	[−.070, −.029]
Spain *(N = 997)*									
Addictive Social Media Use	−.078	.026	[−.129, −.028]	.010	.028	[−.045, .064]	−.088	.015	[−.118, −.059]
Sweden *(N = 1022)*									
Addictive Social Media Use	−.098	.020	[−.138, −.058]	−.007	.022	[−.051, .037]	−.091	.013	[−.116, −.065]
U.K. *(N = 1073)*									
Addictive Social Media Use	−.059	.020	[−.097, −.020]	.009	.023	[−.037, .054]	−.067	.014	[−.095, −.039]
U.S. *(N = 1077)*									
Addictive Social Media Use	−.068	.024	[−.114, −.021]	.042	.024	[−.006, .089]	−.109	.015	[−.138, −.080]

*SE =* standard error, CI=Confidence interval; all CIs generated with bootstrapping: *N* = 10.000

#### Regression Analyses: Relationship between Positive Mental Health, Conscious Physical Activity and Sleep Rhythm during Covid-19 Outbreak

The percentage of individuals who consciously enhanced physical activity during the Covid-19 outbreak ranged between 29.3% (Germany) and 72.6% (China); the percentage of individuals whose sleep rhythm became consciously more regular ranged between 29.8% (the U.K.) and 57.7% (China) (see Table 4). Table 7 displays an overview of the results of the hierarchical regression analyses. The overall adjusted *R*^*2*^ of the regression models ranged between .033 (Germany) and .105 (China). In Step 1, male gender served as a significant statistical predictor of positive mental health in France and in Spain. While belonging to a younger age group was a significant statistical predictor of positive mental health in Russia, belonging to an older age group served as a significant statistical predictor of positive mental health in Germany, Poland, Spain, Sweden, the U.K., and the U.S. Furthermore, marital status (not being single) was a significant statistical predictor of positive mental health in most countries (exception Germany and the U.S.). Moreover, living environment (living in a large city) served as a significant statistical positive mental health predictor in China, but not in the other eight countries. In Step 2, conscious physical activity as well as conscious regular sleep rhythm served as significant statistical predictors of positive mental health in all nine countries (see Table 7). Sleep rhythm was a remarkably stronger statistical predictor than physical activity in China, France, Poland, Russia, and the U.K. In Germany, Spain, Sweden, and the U.S. both predictors showed similar high effects (see Table 7).

**Table 7:** Simplified presentation of hierarchical regression analyses for the nine countries (outcome: positive mental health)

	China	France	Germany	Poland	Russia	Spain	Sweden	U.K.	U.S.
*Step 1* (standardized coefficient beta, *ß*)									
Gender	.007	.062*	.044	.048	.010	.099**	.052	.001	.024
Age group	.050	.017	.077*	121**	−.090**	.133**	.108**	.108**	.266**
Marital status	.077*	.117**	.038	.142**	.091**	.068	.131**	.076*	.062
Living environment	−.111**	.043	.000	.035	−.023	−.014	.003	.008	−.016
*Step 2* (standardized coefficient beta, *ß*)									
Physical activity	.091**	.122**	.099**	.093**	.134**	.117**	.100**	.105**	.136**
Sleep Rhythm	.245**	.172**	.109**	.170**	.221**	.115**	.105**	.171**	.129**
**Overall adjusted** ***R***^***2***^ **(%)**	**10.5**	**7.6**	**3.3**	**9.2**	**8.8**	**6.9**	**6.2**	**6.7**	**12.2**

China: *N* = 1030, France: *N* = 1012, Germany: *N* = 1175, Poland: *N* = 1012, Russia: *N* = 1020, Spain: *N* = 997, Sweden: *N* = 1001, U.K.: *N* = 1073, U.S.: *N* = 1077; gender: 0 = *woman*, 1 = *man*; **p < .01, *p < .05

## Discussion

Due to the need for “social distancing”, the relevance of social media use for daily life has remarkably increased since the Covid-19 outbreak (Depoux et al., 2020; Lemenager et al., 2021). This resulted in an increase of addictive use tendencies (Masaeli & Farhadi, 2021). To assess the potential significance of the tendencies, to identify individuals at risk for them, and to reduce their potential consequences for people’s life (e.g., Brailovskaia et al., 2021a), a universal valid and reliable instrument for the measurement of addictive social media use is urgently required. The present study investigated the psychometric properties of the BSMAS. Our findings provide evidence for the validity and reliability of this instrument in nine countries. Moreover, they reveal positive mental health and sense of control to be potential protective factors against addictive social media use, and they show how positive mental health might be hypothetically enhanced during the pandemic.

The first aim of our study was to investigate the psychometric properties of the BSMAS. In correspondence to earlier investigations from Hungary (Bányai et al., 2017), Iran (Lin et al., 2017), Italy (Monacis et al., 2017), Taiwan and Hong Kong (Leung et al., 2020), we confirmed the unidimensional factor structure of the BSMAS in population representative samples from China, France, Germany, Poland, Russia, Spain, Sweden, the U.K., and the U.S. In all countries, the BSMAS showed good to excellent internal consistency that was similar high as or even higher than in previous studies that investigated student and non-student samples for example in Germany (Brailovskaia et al., 2020a) and in Norway (Andreassen et al., 2017).

Also, the other scale properties were in an acceptable or good range. Item 1 (salience) and Item 2 (tolerance) had the highest scores in the investigated countries, while Item 6 (conflict) had the lowest score. These findings correspond to the theoretical framework of addictive behavior in general and specifically of addictive social media use (Brand et al., 2019; Griffiths, 2005). Notably, especially in the premier stages of the development of addictive tendencies, the positive emotions experienced online can foster cognitive salience of social media use that can result in a permanent thinking of possible online activities (e.g., which photo or written update should be uploaded next) and the wish to implement the thoughts. This can contribute to an intensive social media use and the experience of further positive emotions (Brand et al., 2019; Wegmann et al., 2015). However, over time, the person can develop an ever higher level of tolerance meaning that more and more time has to be spend online to experience the same positive emotions as previously with less time spent online (Longobardi et al., 2020).

Due to the enhanced amount of online time, the individual neglects offline obligations and important dates which consequently can result in interpersonal conflicts at home and at the working place (Tang & Koh, 2017). Previous research reported that the conflicts because of intensive social media use typically develop in the longer-term. They are especially frequent in clinical patients with diagnosed affective disorders who are not able to hide their urge to permanently stay online (Brailovskaia et al., 2019). In the present study, our focus was on the general population. In all nine countries, the mean scores of depression and anxiety symptoms that were assessed by the DASS-21 were within the described normal (not-pathological) range (i.e., subscale depression: normal range up to a score of 9; subscale anxiety: normal range up to a score of 7; Lovibond & Lovibond, 1995). Thus, while salience and tolerance are typically the characteristics of addictive social media use that can be recognized relatively early due to their high level, offline conflicts can become obviously rather in the longer-term and especially in individuals with a low level of mental health who often tend to intensive social media use to escape negative offline experiences (Brailovskaia & Margraf, 2021; Xie & Karan, 2019). These considerations might at least partly explain the pattern of differently high levels of the BSMAS-Items that we found in all nine countries.

The correlation analyses revealed a similar result pattern in the nine countries. In correspondence with previous studies (Andreassen et al., 2016; Brailovskaia et al., 2019; Lin et al., 2017), the scores of the BSMAS were positively linked to the time spent on social media use and to the scores of the DASS-21 subscales depression, anxiety, and stress. These findings confirm the convergent validity of the BSMAS. Earlier research reported addictive social media use to be negatively linked to positive mental health (Brailovskaia et al., 2019) and to sense of control (Brailovskaia & Margraf, 2021). In our study, the scores of the BSMAS were negatively related to the scores of the Sense of Control Scale in all included countries and to the scores of the PMH-Scale in seven of the nine countries (exception: China and Russia). This reveals confirmation of the discriminant validity of the BSMAS.

To sum up, the investigation of the psychometric properties of the BSMAS provided a confirmation of the unidimensional structure of the measure as well as of an adequate to good scale reliability and construct validity of the instrument in nine countries.

The second aim of our study was to assess potential protective factors and mechanisms that are associated with addictive social media use. To achieve this aim, we investigated a mediation model (see Fig. 1). The hypothesized mediation model worked in seven of the nine investigated countries: France, Germany, Poland, Spain, Sweden, the U.K., and the U.S. More specifically, in these countries, addictive social media use was negatively linked to positive mental health (confirmation of Hypothesis 1a) and to sense of control (confirmation of Hypothesis 1b). Positive mental health was positively related to sense of control (confirmation of Hypothesis 1c). Furthermore, sense of control mediated the relationship between positive mental health and addictive social media use (confirmation of Hypothesis 1d). Available research showed that positive mental health can predict the post-treatment severity status and the remission status from anxiety disorders (Teismann et al., 2018), a decrease of adjustment disorder symptoms (Truskauskaite-Kuneviciene et al., 2020), as well as a decrease of suicide-related outcomes (Brailovskaia et al., 2020d). The current results complement previous studies by showing that positive mental health could hypothetically also contribute to the reduction of addictive social media use. But the cross-sectional design of the present study does not allow true causal conclusions.

The Covid-19 associated restrictions of daily life and the uncertainty about the further course of the pandemic can evoke feelings of control loss (Charles et al., 2021) that are typically accompanied by frustrations, anxiety, helplessness, and hopelessness (Skaff, 2007). To at least temporarily escape the psychological burden, some individuals tend to intensive social media use (Cellini et al., 2020). However, this dysfunctional coping-strategy can foster addictive tendencies and negatively impact mental health (Zhao & Zhou, 2021). Following our mediation model, positive mental health could contribute to one’s sense of control which could hypothetically reduce the experience of burden by the Covid-19 situation. Enhanced sense of control confers resilience and can contribute to functional strategies to cope with the stressful situation (Keeton et al., 2008) such as trying to maintain the daily routine as far as possible and adherence to the governmental measures (Brailovskaia & Margraf, 2020; Wright et al., 2021). The functional coping with the stressful Covid-19 situation could foster positive emotions and thus reduce the need to search for them on social media and consequently reduce addictive use tendencies (Brailovskaia & Margraf, 2021).

These considerations contribute to the explanation of the found relationship between positive mental health, sense of control and addictive social media use in seven of the nine investigated countries. In China and Russia, sense of control was negatively associated with addictive social media use (confirmation of Hypothesis 1b). This confirms the assumption that individuals who experience loss of control could tend to problematic online activity. Sense of control was positively linked to positive mental health in both countries (confirmation of Hypothesis 1c). But there was no significant relationship between positive mental health and addictive social media use (contradiction of Hypothesis 1a). Moreover, the mediation model was not significant (contradiction of Hypothesis 1d). Thus, it can be hypothesized that in both countries, positive mental health cannot serve as a protective factor – mediated through sense of control – against addictive social media use which is remarkable. Notably, earlier research from China and Russia emphasized the widespread of addictive social media use among the population, especially among young people (Liu & Ma, 2019; Zotova & Rozanov, 2020). Moreover, recent studies from both countries reported a remarkable increase of problematic social media use since the pandemic outbreak that is negatively linked to mental health (e.g., enhanced level of depression and anxiety symptoms) (Gao et al., 2020; Nekliudov et al., 2020; Zhao & Zhou, 2021; Zhong et al., 2021). Our present results are consistent with the described findings. The sample from China had the highest score of daily time spent on average on social media use (about 3 h 30 min) in comparison to the other investigated countries; the sample from Russia (about 2 h 36 min) had the second highest score (see Table 4). Also, the level of addictive social media use was remarkably high in both countries. Furthermore, the correlation analyses revealed a close positive link between addictive social media use and symptoms of depression, anxiety and stress in China and in Russia (see Table 5). Against the presented background and the not significant meditation model in our current study, we can only speculate – due to the cross-sectional study design – that the negative impact of social media use might be so strong in both countries that positive mental health which is well-known as a significant protective factor (Teismann et al., 2019) cannot provide its positive effect. Notably, there is a large body of studies that focus on risk factors – but not protective factors – of addictive social media use in China (see for example as overview Hussain et al., 2020) and there is overall very little published research on addictive social media use in Russia. Therefore, further experimental longitudinal investigations of factors that might reduce addictive tendencies especially in Russia and in China are urgently required.

The third aim of our study was to identify factors that could contribute to positive mental health especially during the Covid-19 outbreak. Due to the required changes of the daily routine that included enhanced working and studying at home (often via online conferences), reduced traveling, no visits of fitness studios and sport clubs, the lifestyle of many people changed. As showed by recent research, for many individuals it became less healthy. Their physical activity, sleep rhythm and sleep quality worsened, while sedentary behavior increased (Bates et al., 2020; Caputo & Reichert, 2020; Cellini et al., 2020; Chouchou et al., 2021; Eek et al., 2021). The unhealthy lifestyle is positively linked to depression and anxiety symptoms (Chouchou et al., 2021; Evans et al., 2021; Rogowska et al., 2020), and can at least partly explain the decrease of mental health and well-being since the pandemic outbreak (Charles et al., 2021; Galea et al., 2020; Tanaka & Okamoto, 2021).

In contrast, some people consciously strived for a healthier lifestyle since the pandemic outbreak. They engaged in more outdoor physical activity that does not require complex sportive equipment such as jogging and in more indoor activities such as yoga. Furthermore, they consciously regulated their sleep rhythm by fixed bedtime hours (Chouchou et al., 2021; Eek et al., 2021). Our findings provide further cross-national insights in the healthy lifestyle activities during the Covid-19 outbreak. Overall, 45.3% (*n* = 4269) of all participants in the nine countries reported to engage in more physical activity; the sleep rhythm of 39.4% (*n* = 3706) of our participants became consciously more regular. Germany was the country with the lowest percentage scores of physical activity and the U.K. was the country with the lowest percentage scores of regular sleep rhythm. China was the country with the highest scores of both factors (see Table 4). Notably, the results of our Swedish sample considering physical activity (36.6%) correspond to a recently published study from Sweden. In autumn 2020, of 1318 participants, 36% reported an increase of physical activity since the pandemic outbreak (Eek et al., 2021).

As expected, conscious engagement in more physical activity and a conscious regular sleep rhythm during the pandemic were positively associated with positive mental health in the nine countries (confirmation of Hypothesis 2a and of Hypothesis 2b). The present findings extend previous research conducted prior to the pandemic outbreak that emphasized the protective role of physical exercises and a regular sleep rhythm for mental health (Brunborg et al., 2011; Klaperski et al., 2013; Rebar et al., 2015). Our results confirm the cross-national importance of both factors during the extraordinary Covid-19 situation. The fostering of both factors by governmental prevention programs might counteract the reported negative mental health consequences of the pandemic and improve positive mental health. Our findings gain significance when considering that positive mental health is an important protective factor that among others improves the efficacy of therapeutic treatment of mental disorders (Teismann et al., 2018) and reduces suicide-related outcomes (Brailovskaia et al., 2020c, d) that increased during the past year (Tanaka & Okamoto, 2021). Moreover, in France, Germany, Poland, Spain, Sweden, the U.K., and the U.S., positive mental health could hypothetically reduce addictive social media use – as assessed with the BSMAS.

### Limitations and Further Research

Despite the gained knowledge of our investigation that included nine different countries, the following limitations should be taken into account when interpreting our findings. First, due to the cross-sectional design of our study, only hypothetical causality considerations are possible. This is of specific importance considering the investigated mediation model. We cannot exclude other constellations of the investigated variables (e.g., positive mental health or sense of control as outcome). To enable true conclusions on causality, experimental research should replicate our findings. For example, it could be investigated whether an experimental increase of positive mental health by loving-kindness meditation (Totzeck et al., 2020) can foster one’s sense of control and whether this can contribute to the decrease of addictive social media use. Second, in order not to overload the participants, only a limited number of potential covariates were included in the examination of the construct validity of the BSMAS. This is also true for the investigation of potential predictors of positive mental health. Therefore, future studies are suggested to extend our results by the inclusion of further variables. For example, previous research described addictive social media use – as assessed with the BSMAS – to be positively related to other forms of addictive online activity such as Internet Gaming Disorder (Andreassen et al., 2016; Leung et al., 2020), the attachment style (e.g., discomfort with closeness, need for approval) (Monacis et al., 2017), and the personality trait narcissism (Andreassen et al., 2017). In addition, lifestyle factors such as eating behavior, smoking behavior and consume of alcohol could be considering as further predictors of positive mental health (Evans et al., 2021). Third, we assessed the time spent daily on social media use. But we did not assess specific activities that participants engage in during the social media use. This limits potential conclusions on the participants’ pattern of online activity. Previous research described that especially the active use (e.g., uploading of status updates, writing of comments) is linked to the addictive tendencies, while passive use (e.g., reading of other users’ updates) can foster the experience of envy and contributes to depression and anxiety symptoms (e.g., Shaw et al., 2015; Zhao & Zhou, 2021). Therefore, we suggest future research to investigate whether active and/or passive social media use has increased during the pandemic and how this is linked to the addictive tendencies. This could allow a better understanding of the social media use pattern and their association with addictive tendencies. Fourth, due to the very dynamic circumstances, our findings represent a snapshot of the Covid-19 situation in the spring of 2021 in the nine countries studied. Fifth, no African or South American countries were included in the present study, which limits the generalizability of the current findings on the universal psychometric properties of the BSMAS, as well as on the assessed relationships between the investigated variables. Sixth, originally the BSMAS (previously Bergen Facebook Addiction Scale) was developed to assess addictive Facebook use and thus focused on the characteristics of this specific social platform (Andreassen et al., 2012). Many findings on addictive Facebook use were replicated on addictive social media use in general (see e.g., Brailovskaia et al., 2020a). Nevertheless, results gained with the BSMAS should be interpreted with caution.

In summary, the present study provides first representative results from nine different countries in parallel on the evaluation of the psychometric properties of the BSMAS and on potential mechanisms that are associated with addictive social media use during the Covid-19 outbreak. Cross-nationally, our findings show that the BSMAS is a reliable and valid instrument for the assessment of additive social media use. Moreover, we can hypothesize that positive mental health and sense of control might belong to protective factors against the addictive use tendencies across various countries. Specifically, positive mental health could foster sense of control which might reduce addictive social media use. This emphasizes the importance to identify factors that can increase positive mental health especially during the extraordinary Covid-19 situation that is accompanied by an increase of addictive social media use and a decrease of mental health. Conscious physical activity and sleep rhythm regulation could belong to such factors. Their inclusion in protective prevention programs and emphasizing in governmental public communication and through advertising campaigns could be a universal significant step of the fight with negative consequences of the pandemic.

## Data Availability

The dataset and further material analysed during the current study will be available from the corresponding author on reasonable request.
